# Differential Impact of Serotonin Signaling Methylphenidate on Young versus Adult: Insights from Behavioral and Dorsal Raphe Nucleus Neuronal Recordings from Freely Behaving Rats

**DOI:** 10.3390/ijms25158082

**Published:** 2024-07-24

**Authors:** Nachum Dafny, Gloria M. Elizondo, Cruz Perez-Vasquez

**Affiliations:** 1 Department of Neurobiology and Anatomy, McGovern Medical School, University of Texas Health Science Center, Houston, TX 77030, USA; gloria.m.elizondo@uth.tmc.edu; 2 Physiology Department Medical School, National Autonomous University of Mexico, Ciudad de México 04510, Mexico

**Keywords:** age differences, behavior, dorsal raphe nucleus, neuronal activity, serotonin, methylphenidate

## Abstract

Methylphenidate (MPD) remains a cornerstone pharmacological intervention for managing ADHD, yet its increasing usage among ordinary youth and adults outside clinical contexts necessitates a thorough investigation into its developmental effects. This study seeks to simultaneously investigate the behavioral and neuronal changes within the dorsal raphe (DR) nucleus, a center of serotonergic neurons in the mammalian brain, before and after the administration of varying doses of acute and chronic MPD in freely behaving young and adult rats implanted with DR recording electrodes. Wireless neuronal and behavioral recording systems were used over 10 consecutive experimental days. Eight groups were examined: saline, 0.6, 2.5, and 10.0 mg/kg MPD for both young and adult rats. Six daily MPD injections were administered on experimental days 1 to 6, followed by a three-day washout period and MPD re-administration on experimental day 10 (ED10). The analysis of neuronal activity recorded from 504 DR neurons (DRNs) in young rats and 356 DRNs in adult rats reveals significant age-dependent differences in acute and chronic MPD responses. This study emphasizes the importance of aligning electrophysiological evaluations with behavioral outcomes following extended MPD exposure, elucidating the critical role of DRNs and serotonin signaling in modulating MPD responses and delineating age-specific variations in young versus adult rat models.

## 1. Introduction

Attention deficit hyperactivity disorder (ADHD) is a complex behavioral disorder characterized by hyperactivity, increased impulsivity, and inattention [1,2,3]. Methylphenidate (MPD) stands as the most commonly prescribed psychostimulant for managing and treating ADHD [3,4,5,6,7]. Over the past decade, there has been a notable increase in the use of MPD among “ordinary” youths and adults seeking cognitive enhancement to improve intellectual performance in today’s competitive society, alongside instances of its recreational abuse [5,8,9]. Alarmingly, fatalities resulting from MPD use surpass those from cocaine or amphetamines [10]. This surge in MPD consumption among healthy populations has raised concerns about the acute and chronic effects of MPD exposure on individuals without ADHD [5,11,12,13], although the precise neurophysiological mechanisms of its action remain elusive [14,15,16,17,18,19,20].

While research on ADHD has traditionally centered on dopamine (DA) and norepinephrine (NE) signaling in its pathophysiology [14,16,17,20,21,22], emerging evidence implicates serotonin (5-HT) transmission in behavioral disorders [23], suggesting a potential role of serotonin in ADHD symptoms [24,25,26]. Behavioral and histochemical studies suggest that MPD also influences serotonin (5 HT) levels [27]. Given that the DRNs are a major source of 5-HT in the mammalian brain (Baker et al., 1991; Barnes and Sharp, 1999; Kuczenski and Segal, 1999) [28,29,30] and their neuronal signaling correlates with various behaviors, including reward and reinforcement [15,31], it is plausible that MPD’s efficacy partially stems from its impact on the DRN and 5-HT systems [32]. MPD exposure alters both the 5-HT transporter and 5-HT levels [33,34], resulting in a calming effect on ADHD patients. Consequently, the DR nucleus was chosen as the focal point of this study.

Past research has shown that psychostimulants such as cocaine and amphetamine exert dose-dependent effects on serotonergic DRNs in anesthetized rats [35,36]. However, there are limited electrophysiological and behavioral studies investigating the acute and chronic effects of MPD exposure on DRNs and animals’ behaviors. Thus, this study employed unanesthetized, freely behaving rats implanted with permanent electrodes bilaterally in the DR nucleus. Repetitive MPD exposure has been linked to elicit behavioral withdrawal, sensitization, or tolerance [15,19,37,38]. Behavioral sensitization or tolerance refers to the increased or decreased response, respectively, to repeated drug exposure compared to the initial drug effect [15,19,38,39]. It was reported that young and adult rats exhibit different behavioral responses to acute and chronic psychostimulant exposure, with chronic exposure in young rats inducing long-term depression-like behavior in adults [40,41,42]. Other studies have shown that psychostimulant use during childhood does not significantly alter the cortical mantle, and MPD users with ADHD exhibit functional attenuation similar to non-users [43,44]. Despite its widespread use, the long-term effects of repetitive (chronic) MPD exposure on young brain development remain poorly understood [45,46,47]. While the dopaminergic and glutaminergic signaling systems have established roles in the neuroplasticity underlying psychostimulant action [22,48], there is a scarcity of studies on the serotonergic signaling involved in the neurophysiological mechanism of MPD and other psychostimulants [30,49,50].

Previous studies have demonstrated that MPD induces both behavioral and DR neuronal changes in adult rats [51,52] and young rats [53]. However, there is a gap in the literature comparing DR neuronal activity responses to acute and chronic MPD exposure in these two age populations. This study hypothesizes that the same MPD dose will elicit behavioral sensitization in some young and adult rats and behavioral tolerance in others. Moreover, the ratio of how many young rats’ express sensitization or tolerance will significantly differ from adult rats. Additionally, DRN activity recorded from behaviorally sensitized rats is expected to significantly differ from that of behaviorally tolerant rats, and there will be significant age differences in the number of young rats expressing sensitization or tolerance compared to adult rats. The objective of this study is to test the following hypotheses: (1) The chronic administration of 0.6, 2.5, or 10.0 mg/kg of MPD will lead to behavioral and neuronal sensitization in some animals, while others receiving the same MPD dose will exhibit behavioral and neurophysiological tolerance when compared to the initial effects of the drug for each dose. (2) Neuronal activity recorded from animals displaying behavioral sensitization to chronic MPD exposure will primarily show excitation in response to MPD treatment. In contrast, neuronal activity from animals showing behavioral tolerance will primarily demonstrate a decrease in firing rate in response to chronic MPD treatment compared to the acute (initial) effects of MPD. (3) Adolescent animals will respond differently to 0.6, 2.5, or 10.0 mg/kg of MPD compared to adult animals. Specifically, the ratio of LC neurons responding to each MPD dose with excitation or attenuation will differ significantly between age groups, indicating an age-related difference in response to MPD. Thus, the aim of this study is to employ an acute and chronic dose–response protocol of MPD to investigate potential age differences in behavioral and DRN properties between young and adult rats.

## 2. Results

### 2.1. Locomotor Behavior of Time and Saline Control

**Figure 1:**
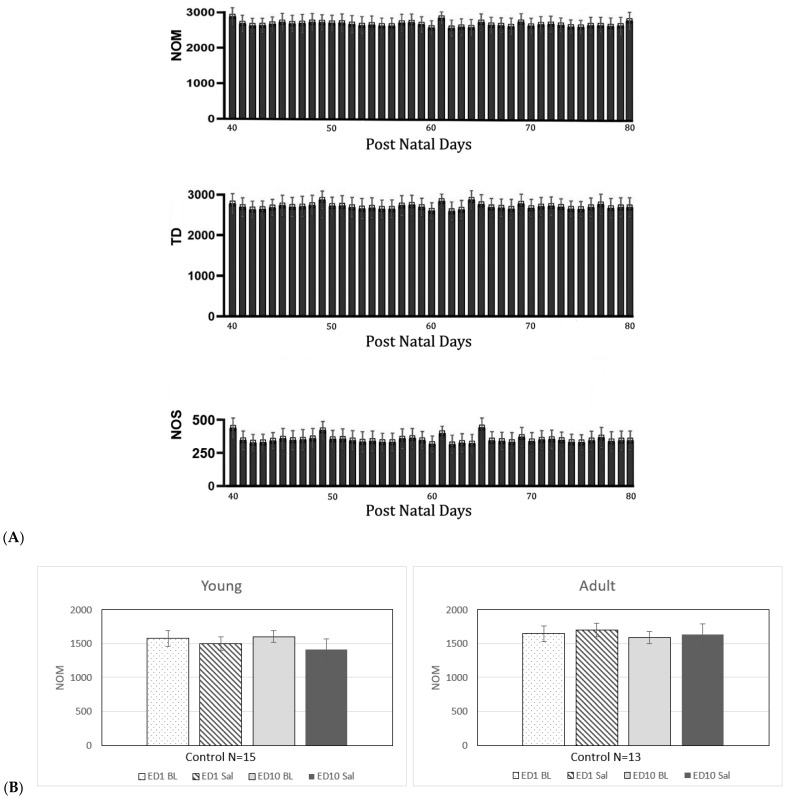
Time and saline control. (**A**) (N = 8). The figure summarizes the number of movements (NOM), total traveling (TD) in cm, and the number of stereotypic (NOS) movements over 40 sequential days from post-natal day 40 to 80 showing that the locomotor behavioral activities during the 40 days exhibited similar activities. (**B**) summarizes the saline control groups of young (N = 15) and adult (N = 13) male rats. Number of movements (NOM) summarizes the total number of movement activities. During the ten recording days, both age groups exhibit similar activities with no minor fluctuation, indicating that animal handling and injection during the ten experimental days are similar.

To investigate the effects of growing days, injection volume, and animal handling on locomotor activity, two groups of rats were used at each age: a time control young and adult group, each N = 8, and a saline control group (N = 15 for young rats and N = 13 for adult rats) (Figure 1). In un-injected young and adult rats, time control locomotor activity was recorded over 40 consecutive days. Throughout these 40 days, consistent levels of locomotor activity were observed (Figure 1A, time control). Similar stability in locomotor activity was noted in the saline control group (Figure 1B). Locomotor activity readings (NOM, TD, and NOS) exhibited non-significant differences with minor fluctuations across recording days and subsequent acute and repetitive saline administrations. This suggests that the locomotor behavioral activities (NOM, TD, and NOS) recorded remain unaffected by 40 days (P-40 to P-80) of growing, the injection process, animal handling, or laboratory conditions. Since there were no significant differences in locomotor behavioral activity between daily activity (Figure 1A) and following saline injections (Figure 1B), the locomotor activities observed on ED1 following saline were used as a control (ED1 BL) for comparison against MPD injection groups. Thus, any significant changes in locomotor activity from the ED1 BL activity can be attributed to the effects of MPD exposure.

### 2.2. Experimental Rat Groups

In total, 178 young rats received acute and repetitive injections of saline, 0.6, 2.5, or 10.0 mg/kg MPD as follows: 15, 55, 51, and 57, respectively. Additionally, 146 adult rats received acute and repetitive injections of saline, 0.6, 2.5, and 10.0 mg/kg MPD, respectively, as follows: 13, 45, 41, and 47.

### 2.3. Behavioral Response to Acute and Chronic 0.6 mg/kg MPD (Figure 2A,D)

**Figure 2:**
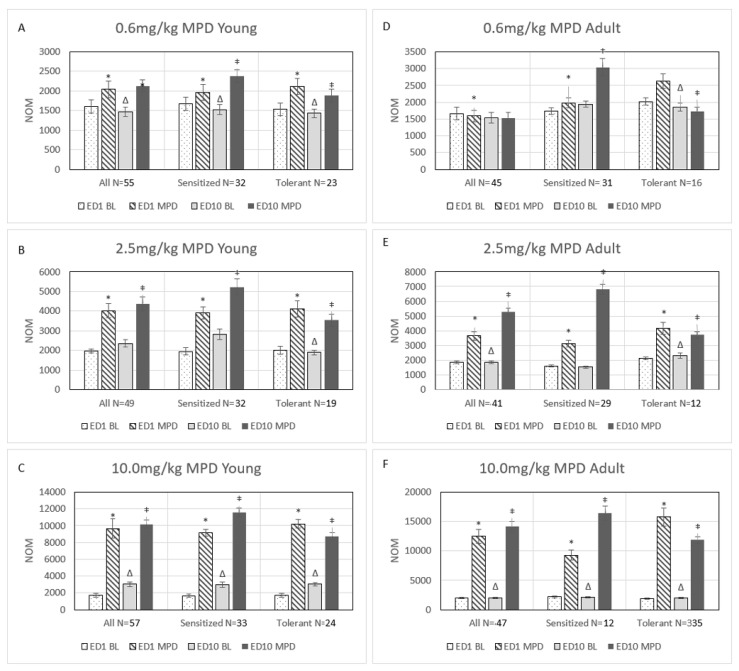
Summary of the behavioral data (number of movements, NOM) for young and adult rat groups following 0.6 mg/kg MPD in (**A**,**D**), following 2.5 mg/kg MPD in (**B**,**E**), and following 10.0 mg/kg MPD in (**C**,**F**). Each histogram is labeled from (**A**–**C**) for the young age group and (**D**–**F**) for the adult age group. The rats in the experimental MPD dose groups were divided into 3 subgroups: all animals, behaviorally sensitized animals, and behaviorally tolerant animals. The “all” group summarizes all the animals for the respective MPD dose. The “sensitized” group and “tolerant” group summarize only animals that expressed either behavioral sensitization or tolerance to chronic MPD at ED10 after six daily MPD exposures (0.6, 2.5, 10.0 mg/kg) and three washout days (ED7, 8, 9), as compared to the initial MPD exposure at ED1, respectively. Each histogram contains four columns, ED1 BL, ED1 MPD, ED10 BL, and ED10 MPD, organized into three comparisons per subgroup: ED1 MPD/ED1 BL, to obtain the MPD acute effect; ED10 BL/ED1 BL, to obtain the effect of six daily MPD exposures and three washout days on ED10 BL which will indicate if withdrawal behavior is expressed; and ED10 MPD/ED1 MPD, to obtain the chronic MPD effect, sensitization, and tolerance. The NOM of young ED1 MPD is compared to the NOM of adult ED1 MPD to examine the behavioral difference between the acute response to MPD for young and adult rats; the NOM of young ED10 MPD is compared to the NOM of adult ED10 MPD to examine the difference in behavior in response to chronic MPD in young and adult rats. Above each column is the standard deviation (SD). *—indicates significant (*p* < 0.05) differences from ED1 BL (ED1 BL/ED1MPD, acute). ∆—indicates significant (*p* < 0.05) differences from ED1 BL to ED10 BL (ED1 BL/ED10 BL, withdrawal). ‡—indicates significant (*p* < 0.05) differences from ED1 MPD (ED1 MPD/ED10 MPD, chronic).

Fifty-five young and forty-five adult rats received acute and chronic injections of 0.6 mg/kg MPD. Both age groups of rats exhibited a significant increase in their locomotive behavioral activity (*p* < 0.05) following the acute administration of 0.6 mg/kg MPD on ED1 (ED1 MPD/ED1 BL). After six daily MPD exposures and three washout days, young animals exhibited a significant (*p* < 0.05) increase in their locomotor activity, while the adult rats exhibited no change (ED10 BL/ED1 BL). MPD rechallenge at ED10 caused a significant (*p* < 0.05) increase in locomotion only in the adult rats group. This increase in activity is considered an expression of behavioral sensitization (Figure 2A,D).

### 2.4. Behavioral Sensitization Group in Response to 0.6 mg/kg MPD (Figure 2A,D, Sensitized Group)

When animals were categorized into subgroups based on their individual behavioral response to MPD on ED10 compared to ED1 MPD using the CR test and the Student’s *t*-test, 32 young rats and 31 adult rats exhibited a significant increase (*p* < 0.05) in locomotor activity following MPD rechallenge as compared to MPD acute effects (ED10 MPD/ED1 MPD). These animals were classified as demonstrating significant (*p* < 0.05) behavioral sensitization. Both young and adult rats also showed a significant (*p* < 0.05) increase in locomotor activity on ED1 following the acute administration of MPD (ED1 MPD/ED1 BL). However, only the young rats in the sensitization group demonstrated a significant (*p* < 0.05) change in ED10 BL compared to ED1 BL (ED10 BL/ED1 BL), indicating withdrawal. The ratio of young rats exhibiting behavioral sensitization to 0.6 mg/kg MPD was 58.2% (32 out of 55), and for adults, the ratio showing behavioral sensitization to 0.6 mg/kg MPD was 68.9% (31 out of 45).

### 2.5. Behavioral Tolerance Group in Response to 0.6 mg/kg MPD (Figure 2A,D, Tolerant Group)

Twenty-three young rats and fourteen adult rats exhibited behavioral tolerance to repetitive 0.6 mg/kg MPD compared to the initial MPD effects (ED10 MPD/ED1 MPD). Both age groups demonstrated a significant decrease (*p* < 0.05) in locomotor activity following MPD rechallenge on ED10 when compared to activity post MPD injection on ED1 (ED10 MPD/ED1 MPD). These animals were classified as expressing behavioral tolerance. Both young and adult rats demonstrated a significant (*p* < 0.05) increase in locomotor behavior following acute 0.6 mg/kg MPD administration on ED1. Additionally, both age groups showed significant changes in ED10 BL/ED1 BL activity (*p* < 0.05). The ratio of young rats showing behavioral tolerance to 0.6 mg/kg MPD was 41.8% (23 out of 55), and for adult rats, it was 31.1% (14 out of 45). A comparison of these age ratios using the chi-square test resulted in a significant (*p* < 0.05) difference between the two age groups regarding 0.6 mg/kg MPD.

### 2.6. Behavioral Response to Acute and Chronic Administration of 2.5 mg/kg MPD (Figure 2B,E, All Groups)

Fifty-one young and forty-one adult rats received acute and chronic injections of 2.5 mg/kg MPD. When analyzed as whole populations (all groups), both young and adult rats demonstrated a significant (*p* < 0.05) increase in behavioral response following the acute administration of MPD on ED1 (ED1 MPD/ED1 BL). The ED10 BL activity after six daily exposures to MPD and three washout days (ED10 BL/ED1 BL) did not significantly change in the young rat group, while it significantly (*p* < 0.05) increased in the adult rat group. Following MPD rechallenge at ED10, both age groups of rats demonstrated a significantly (*p* < 0.05) increased behavioral response compared to the initial response to MPD on ED1 (ED10 MPD/ED1 MPD).

### 2.7. Behavioral Sensitization to Chronic Administration of 2.5 mg/kg MPD (Figure 2B,E, Sensitized Groups)

Thirty-two young rats and twenty-nine adults exhibited behavioral sensitization to chronic MPD exposure. Both age groups displayed a significant (*p* < 0.05) increase in locomotor activity following acute MPD administration (ED1 MPD/ED1 BL), with no difference between ED10 BL and ED1 BL after six daily administrations of 2.5 mg/kg MPD and three washout days. Following MPD rechallenge at ED10, both young and adult rats demonstrated a significantly (*p* < 0.05) further increased behavioral response to MPD compared to the response to MPD on ED1 (ED10 MPD/ED1 MPD). The ratio of young rats showing behavioral sensitization to chronic 2.5 mg/kg MPD was 62.7% (32 out of 51). The ratio of adult rats showing behavioral sensitization to 2.5 mg/kg MPD was 70.7% (29 out of 41).

### 2.8. Behavioral Tolerance to Chronic 2.5 mg/kg MPD (Figure 2B,E, Tolerant Groups)

Nineteen young and twelve adult rats exhibited behavioral tolerance to chronic MPD exposure. Both age groups displayed a significant (*p* < 0.05) increase in locomotor activity following acute MPD administration on ED1 (ED1 MPD/ED1 BL). Young and adult rats had a significant (*p* < 0.05) change in locomotor activity from ED1 BL to ED10 BL, with young rats exhibiting a decrease in activity and adults exhibiting an increase in activity (ED10 BL/ED1 BL). Both groups demonstrated a significant (*p* < 0.05) decrease in locomotor activity following MPD rechallenge on ED10 compared to activity with MPD on ED1 (ED10 MPD/ED1 MPD). The ratio of young rats showing behavioral tolerance to 2.5 mg/kg MPD was 37.3% (19 out of 51). The ratio of adult rats showing behavioral tolerance to 2.5 mg/kg MPD was 29.3% (12 out of 41).

### 2.9. Behavioral Response to Acute and Chronic 10.0 mg/kg MPD (Figure 2C,F, All Groups)

Fifty-seven young and forty-seven adult rats received acute and chronic injections of 10.0 mg/kg MPD. When analyzed as whole populations, both young and adult rats demonstrated a significant increase in behavioral activity (*p* < 0.05) following the acute administration of 10 mg/kg MPD on ED1 (ED1 MPD/ED1 BL). Both age groups exhibited a significant (*p* < 0.05) increase in locomotor activity on ED 10 compared to ED1 BL (ED10 BL/ED1 BL). However, the young animals exhibited a significant (*p* < 0.05) increase in ED10 BL activity compared to adult ED10 BL. Following rechallenge at 10.0 mg/kg MPD, both age groups of rats demonstrated a significantly (*p* < 0.05) increased behavioral response on ED10 (ED10 MPD/ED1 MPD).

### 2.10. Behavioral Sensitization Group, 10.0 mg/kg MPD (Figure 2C,F, Sensitized Groups)

Thirty-three young and twelve adult rats exhibited behavioral sensitization to chronic 10 mg/kg MPD exposure. The behaviorally sensitized rats in both age groups displayed a significant (*p* < 0.05) increase in locomotor activity following acute 10 mg/kg MPD administration (ED1 MPD/ED1 BL) and a significant (*p* < 0.05) further increase in locomotor activity following MPD rechallenge on ED10 (ED10 MPD/ED1 MPD). Behaviorally sensitized young and adult rats demonstrated a significant (*p* < 0.05) increase in ED10 BL/ED1 BL, while the young group exhibited significantly (*p* < 0.05) less locomotor activity compared to the adult rats group. The ratio of young rats showing behavioral sensitization to 10 mg/kg MPD is 57.9% (33 out of 57). The ratio of adult rats showing behavioral sensitization to 10 mg/kg MPD is 25.5% (12 out of 47). A comparison of these age ratios using the chi-square test resulted in a significant (*p* < 0.01) difference between the two age groups regarding 10.0 mg/kg MPD.

### 2.11. Behavioral Tolerance Group, 10.0 mg/kg MPD (Figure 2C,F, Tolerance Groups)

Twenty-four young and thirty-five adult rats demonstrated behavioral tolerance to chronic 10 mg/kg MPD exposure. The behaviorally tolerant groups of both age groups displayed a significant (*p* < 0.05) increase in locomotor activity following acute MPD administration (ED1 MPD/ED1 BL), while the young rats group exhibited significantly (*p* < 0.05) less locomotor activity than the adult rats group. A significant (*p* < 0.05) decrease in locomotor activity following rechallenge of MPD on ED10 (ED10 MPD/ED1 MPD) was observed in both age groups. Both age groups had a significant (*p* < 0.05) increase in ED10 BL activity compared to ED1 BL (ED10 BL/ED1 BL), expressing withdrawal. The ratio of young rats showing behavioral tolerance to 10.0 mg/kg MPD is 42.1% (24 out of 57). The ratio of adult rats showing behavioral tolerance to 10.0 mg/kg MPD is 74.5% (35 out of 47). The 10.0 mg/kg MPD dose is the only dose at which the majority of the adult rats became behaviorally tolerant to the effects of MPD.

### 2.12. Comparing the Number of Movements (NOM) between the Three Groups: All, Sensitized, and Tolerance

Comparing the effect of acute doses of 0.6, 2.5, and 10 mg/kg MPD (ED1 MPD/ED1 BL), the change in ED10 BL/ED1 BL after six daily MPD administrations and three washout days, and the effect of chronic MPD exposure (ED10 MPD/ED1 MPD) between the two age groups reveals a significant (*p* < 0.001) difference in response to MPD between young and adult animals using the chi-square test.

### 2.13. Dorsal Raphe Neuron (DRN) Activity (Table 1 and Table 2)

Five hundred and four DRNs were evaluated from young rats, and 356 DRNs were evaluated from adult rats; all DRNs were histologically confirmed to be recorded from the DR nucleus. All neuronal recordings in this study exhibited similar waveform spikes and amplitudes on ED10 compared to ED1.

**Table 1:** The table summarizes the DR neuron (DRN) responses following 0.6, 2.5, and 10.0 mg/kg MPD recorded from young animals. In 1A is the summary from the DRN responses recorded from all the animals, in 1B from animals expressing behavioral sensitization, and in 1C from animals expressing behavioral tolerance. Under acute, baseline, and chronic are how many DRNs responded significantly (*p* < 0.05) and their % (in bracket) response to each MPD dose (0.6, 2.5, or 10.0 mg/kg) by excitation (arrow up) or attenuation (arrow down) and the number and % neuronal DRNs that did not respond to MPD following acute MPD (ED1 MPD/ED1 BL). The BL activity of ED10 compares to ED1 BL (ED10 BL/ED1 BL) as well the MPD chronic effect of each MPD dose (ED10 MPD/ED1 MPD).

**A**	**All Animals**										
	**Dose**	**N**	**Acute** **(ED 1 MPD/ED 1 BL)**			**Baseline** **(ED 10 BL/ED 1 BL)**			**Chronic** **(ED 10 MPD/ED 1 MPD)**		
			⇑	⇓	⇔	⇑	⇓	⇔	⇑	⇓	⇔
	Saline	57	1 (1.8%)	1 (1.8%)	55 (96.5%)	3 (5.3%)	2 (3.5%)	52 (91.2%)	2 (3.5%)	1 (1.7%)	54 (94.7%)
	0.6 mg/kg	137	35 (26%)	32 (23%)	70 (51%)	39 (29%)	26 (19%)	72 (52%)	26 (19%)	40 (29%)	71 (52%)
	2.5 mg/kg	142	53 (37%)	45 (32%)	44 (31%)	49 (35%)	36 (25%)	57 (40%)	48 (34%)	48 (34%)	46 (32%)
	10.0 mg/kg	168	80 (48%)	65 (39%)	23 (13%)	72 (43%)	63 (38%)	33 (19%)	73 (44%)	66 (39%)	29 (17%)
	Total	504									
**B**	**Sensitized**										
	**Dose**	**N**	**Acute** **(ED 1 MPD/ED 1 BL)**			**Baseline** **(ED 10 BL/ED 1 BL)**			**Chronic** **(ED 10 MPD/ED 1 MPD)**		
			⇑	⇓	⇔	⇑	⇓	⇔	⇑	⇓	⇔
	0.6 mg/kg	70	43 (61%)	20 (29%)	7 (10%)	33 (47%)	27 (39%)	10 (14%)	42 (60%)	22 (31%)	6 (9%)
	2.5 mg/kg	62	49 (79%)	9 (15%)	4 (6%)	44 (71%)	11 (18%)	7 (11%)	48 (77%)	11 (18%)	3 (5%)
	10.0 mg/kg	109	73 (67%)	30 (28%)	6 (5%)	53 (49%)	37 (34%)	19 (17%)	67 (62%)	35 (32%)	7 (6%)
	Total	241									
**C**	**Tolerance**										
	**Dose**	**N**	**Acute** **(ED 1 MPD/ED 1 BL)**			**Baseline** **(ED 10 BL/ED 1 BL)**			**Chronic** **(ED 10 MPD/ED 1 MPD)**		
			⇑	⇓	⇔	⇑	⇓	⇔	⇑	⇓	⇔
	0.6 mg/kg	67	14 (21%)	17 (25%)	36 (54%)	15 (23%)	13 (19%)	39 (58%)	10 (15%)	28 (42%)	29 (43%)
	2.5 mg/kg	80	14 (17%)	36 (45%)	30 (38%)	19 (24%)	22 (28%)	39 (48%)	12 (15%)	34 (46%)	31 (39%)
	10.0 mg/kg	59	11 (19%)	34 (58%)	14 (23%)	22 (37%)	18 (31%)	19 (32%)	6 (10%)	28 (48%)	25 (42%)
	Total	206									

**Table 2:** The table summarizes the DRN responses following 0.6, 2.5, and 10.0 mg/kg MPD recorded from adult animals. In A, B, and C are the summaries of the DRN responses recorded from all the animals (2A), from animals expressing behavioral sensitization (2B), and from animals expressing behavioral tolerance (2C), respectively. Under acute, baseline, and chronic are how many DRNs responded significantly (*p* < 0.05) and their % (in bracket) response to each MPD dose (0.6, 2.5, or 10.0 mg/kg) by excitation (arrow up) or attenuation (arrow down) and the number and % neuronal DRNs that did not respond to MPD following acute MPD (ED1 MPD/ED1 BL). The baseline (BL) activity of ED10 compares to ED1 BL (ED10 BL/ED1 BL) as well the MPD chronic effect of each MPD dose (ED10 MPD/ED1 MPD).

**A**	**All Animals**										
	**Dose**	**N**	**Acute** **(ED 1 MPD/ED 1 BL)**			**Baseline** **(ED 10 BL/ED 1 BL)**			**Chronic** **(ED 10 MPD/ED 1 MPD)**		
			⇑	⇓	⇔	⇑	⇓	⇔	⇑	⇓	⇔
	Saline	27	0	0	27 (100%)	1 (4%)	2 (7%)	24 (89%)	1 (4%)	1 (4%)	25 (92%)
	0.6 mg/kg	122	39 (32%)	19 (16%)	64 (52%)	51 (42%)	57 (47%)	14 (11%)	48 (39%)	58 (48%)	16 (13%)
	2.5 mg/kg	94	49 (52%)	5 (5%)	40 (43%)	35 (37%)	32 (35%)	27 (28%)	45 (48%)	29 (31%)	20 (21%)
	10.0 mg/kg	113	72 (64%)	11 (10%	30 (26%)	37 (33%)	61 (54%)	15 (13%)	39 (35%)	61 (54%)	13 (11%)
	Total	356									
**B**	**Sensitized**										
	**Dose**	**N**	**Acute** **(ED 1 MPD/ED 1 BL)**			**Baseline** **(ED 10 BL/ED 1 BL)**			**Chronic** **(ED 10 MPD/ED 1 MPD)**		
			⇑	⇓	⇔	⇑	⇓	⇔	⇑	⇓	⇔
	0.6 mg/kg	27	5 (19%)	6 (19%)	16 (59%)	11 (41%)	9 (33%)	7 (26%)	10 (37%)	5 (19%)	12 (44%)
	2.5 mg/kg	42	17 (40%)	1 (3%)	24 (57%)	13 (31%)	22 (53%)	7 (16%)	20 (48%)	17 (40%)	5 (12%)
	10.0 mg/kg	20	15 (75%)	0 (0.0%)	5 (25%)	4 (20%)	15 (75%)	1 (5%)	3 (15%)	16 (80%)	1 (5%)
	Total	89									
**C**	**Tolerance**										
	**Dose**	**N**	**Acute** **(ED 1 MPD/ED 1 BL)**			**Baseline** **(ED 10 BL/ED 1 BL)**			**Chronic** **(ED 10 MPD/ED 1 MPD)**		
			⇑	⇓	⇔	⇑	⇓	⇔	⇑	⇓	⇔
	0.6 mg/kg	95	33 (35%)	13 (14%)	49 (51%)	38 (40%)	49 (52%)	8 (8%)	39 (41%)	54 (57%)	2 (2%)
	2.5 mg/kg	52	33 (64%)	4 (8%)	15 (28%)	21 (40%)	11 (21%)	20 (39%)	23 (44%)	14 (27%)	15 (29%)
	10.0 mg/kg	93	59 (63%)	10 (11%)	24 (26%)	32 (34%)	46 (50%)	15 (16%)	36 (39%)	45 (48%)	12 (13%)
	Total	240									

### 2.14. Saline Control Recording (Table 1A and Table 2A)

Fifty-seven and twenty-seven DRNs were recorded from young and adult rats, respectively, as controls following acute and repetitive saline injections (Table 1A and Table 2A). Of the fifty-seven DRNs recorded from young rats, one neuron showed an increase in neuronal activity following the acute injection of saline on ED1, and one neuron showed a decrease in neuronal activity, while fifty-five neurons showed no change in neuronal activity (ED1 Sal/ED1 BL). Three neurons showed an increase in baseline neuronal activity, two neurons showed a decrease in baseline neuronal activity, and fifty-two neurons did not show a change in baseline neuronal activity on ED10 (ED10 BL/ED1 BL). Upon rechallenge with saline on ED10 (ED10 Sal/ED1 Sal), two neurons showed an increase in neuronal activity, while one neuron showed a decrease in neuronal activity, and fifty-four showed no change in neuronal activity upon saline rechallenge (Table 1A, saline). Of the 27 DRNs recorded from adults, none showed any significant changes in neuronal activity following the initial injection of saline on ED1 (ED1 Sal/ED1 BL). One DRN recorded showed an increase in baseline activity, two neurons showed a decrease in baseline activity, and twenty-four neurons showed no change in baseline activity on ED10 BL/ED1 BL. Following saline rechallenge on ED10 (ED10 Sal/ED1 Sal), one DRN showed an increase in neuronal activity, while one neuron showed a decrease in neuronal activity, and twenty-five neurons showed no change in activity (Table 2A, saline). These observations indicate that the few DRNs that exhibit alterations in ED1 BL activity following saline injection were random and that the saline injections, animal handling, and laboratory conditions did not have a significant effect on neuronal activity in DRNs in both age groups. Therefore, any significant changes in neuronal recordings from ED1 saline (i.e., ED1 BL) are presumably due to the effects of MPD exposure.

### 2.15. Impact of 0.6 mg/kg MPD on DRNs Recorded from All Animal Groups (Table 1A and Table 2A)

One hundred and thirty-seven DRNs were recorded from all groups of young rats (Table 1A). Of these neurons, at ED1 MPD/ED1 BL, 47% (67/137) responded with a significant (*p* < 0.05) change in neuronal firing activity following the acute administration of MPD on ED1, with the majority (52%; 35/67) showing an increase in firing rate. When comparing ED10 BL/ED1 BL neuronal activity, 48% (65/137) showed a significant (*p* < 0.05) change in their neuronal firing rate at ED10 BL/ED1 BL. Among these neurons, the majority (60%; 39/65) showed a significant (*p* < 0.05) increase in firing rate, while 40% (26/65) demonstrated a significant (*p* < 0.05) decrease in firing rate. Upon rechallenge of MPD on ED10, 48% (66/137) responded with a significant (*p* < 0.05) change in neuronal firing rate. Among these responding neurons, 39% (26/66) responded with a significant (*p* < 0.05) increase in firing rate, while 61% (40/66) responded with a significant decrease in firing rate (Table 1A, 0.6 mg/kg MPD, all groups).

A total of 122 DRNs’ activities following acute and chronic administration of 0.6 mg/kg MPD were recorded from adult animals (Table 2A). Among these DRNs, 48% (58/122) showed a significant (*p* < 0.05) change in their neuronal firing rate following the acute administration of MPD on ED1 MPD/ED1 BL, with the majority of them showing a significant (*p* < 0.05) increase in firing rate (Table 2A, all groups). Of these 122 DRNs at ED10 BL/ED1 BL, 89% (108/122) showed a significant (*p* < 0.05) change, with an even split of 47% (51/108) showing an increase in firing rate and 53% (57/108) showing a decrease in activity after six daily doses of 0.6 mg/kg MPD (ED1 to ED6) and three washout days (ED7 to ED9). Following MPD rechallenge on ED10 (ED10 MPD/ED1 MPD), 87% (106/122) responded with a significant (*p* < 0.05) change in neuronal activity. Among these DRNs responding, 45% (48/106) responded with a significant (*p* < 0.05) increase in activity, while 55% (58/106) responded with a significant decrease in activity (Table 2A, 0.6 mg/kg MPD, all groups).

### 2.16. Impact of 0.6 mg/kg MPD on DRNs Recorded from Animals Expressing Behavioral Sensitization to Chronic 0.6 mg/kg MPD (Table 1B and Table 2B)

Seventy DRNs were recorded from young animals that demonstrated behavioral sensitization to chronic exposure to 0.6 mg/kg MPD (Table 1B). Of these responding neurons at ED1 MPD/ED1 BL, 90% (63/70) responded with a significant (*p* < 0.05) change in firing rate, with 61% (43/70) demonstrating an increase in firing rate. When comparing ED10 BL/ED1 BL, 86% (60/70) of DRNs responded with a significant (*p* < 0.05) change in firing rate. Of these neurons, a majority 55% (33/60) exhibited a significant increase in firing rate. Upon rechallenge of MPD on ED10 (ED10 MPD/ED1 MPD), 91% (64/70) of DRNs responded with a significant (*p* < 0.05) change in their firing rate. Of these neurons, a majority, 65% (42/64), responded to 0.6 mg/kg MPD with an increase in firing rate (Table 1B, 0.6 mg/kg MPD, sensitized).

Twenty-seven DRNs were recorded from adult animals that demonstrated behavioral sensitization to chronic exposure to 0.6 mg/kg MPD (Table 2B). Of these neurons at ED1 MPD/ED1 BL, 41% (11/27) showed a significant (*p* < 0.05) change in firing rate, with 45% (5/11) demonstrating an increase in firing rate. Of these DRNs at ED10 BL/ED1 BL, 74% (20/27) of them exhibited a significant (*p* < 0.05) difference in firing rates after six daily 0.6 mg/kg MPD exposures and three washout days on ED10 BL. Of these neurons, 55% (11/20) exhibited an increase in firing rate at ED10 MPD/ED1 MPD following MPD rechallenge (ED10 MPD/ED1 MPD). Fifty-six percent (15/27) of the DRNs responded with a significant (*p* < 0.05) change in neuronal firing rate. A majority, 67% (10/15), responded with a significant (*p* < 0.05) increase in firing rate (Table 2B, 0.6 mg/kg MPD, sensitized group).

### 2.17. Impact of 0.6 mg/kg MPD on DRN Activity Recorded from Animals Expressing Behavioral Tolerance to Chronic 0.6 mg/kg MPD (Table 1C and Table 2C)

Recordings of neuronal activity from animals exhibiting behavioral tolerance to chronic administration of 0.6 mg/kg MPD revealed significant findings. Sixty-seven neurons from the DRNs were recorded in young animals displaying behavioral tolerance to chronic MPD administration (Table 1C). Among these neurons, 46% (31/67) exhibited a significant (*p* < 0.05) alteration in firing rate following acute MPD injection. Specifically, 45% (14/31) demonstrated an increase in firing rate, while 55% (17/31) showed a decrease. A comparison of the neuronal activity between ED10 BL and ED1 BL after six consecutive days of MPD exposure and three washout days revealed significant changes in 42% (28/67) of the recorded neurons. Among these, 54% (15/28) displayed an increase in firing rate. Upon rechallenge with MPD on ED10 MPD/ED1 MPD, 57% (38/67) of the neurons exhibited a significant (*p* < 0.05) change in firing rate. Among these DRNs, the majority (74%, 28/38) demonstrated a decrease, while 26% (10/38) displayed a significant increase in firing rate (Table 1C, 0.6 mg/kg MPD, tolerance group).

Recordings from adult animals expressing behavioral tolerance to chronic 0.6 mg/kg MPD (Table 2C) revealed notable patterns. Ninety-five DRNs were recorded from adult rats, with 48% (46/95) showing a significant (*p* < 0.05) change in neuronal firing rate following acute MPD injection. Among these, the majority (72%, 33/46) displayed an increase in firing rate. Comparing BL activity at ED10 BL to ED1 BL revealed significant (*p* < 0.05) age differences in 92% (87/95) of the recorded DRNs from adults. Among these, 44% (38/87) exhibited an increase in the baseline neuronal firing rate, while 56% (49/87) showed a decrease. Upon rechallenge with MPD on ED10 MPD/ED1 MPD, 98% (93/95) of the recorded DRNs from adults exhibited a significant (*p* < 0.05) change in firing rate. Among these, the majority (58%, 54/93) displayed a decrease in firing rate (Table 2C, 0.6 mg/kg MPD, tolerance group), while the young rats exhibited a different ratio (Table 1C, tolerance). These findings underscore the impact of chronic MPD exposure on neuronal activity and the development of behavioral tolerance, with distinct patterns observed in both young and adult animals.

### 2.18. Impact of 2.5 mg/kg MPD on DRN Activity Was Recorded from All the Animals (Table 1A and Table 2A)

One hundred and forty-two DRNs were recorded from young animals (Table 1A). Among these neurons, 69% (98/142) responded with a significant (*p* < 0.05) change in their neuronal firing activity following the acute administration of MPD, with 54% (53/98) showing an increase in firing rate. When comparing ED10 BL/ED1 BL, 60% (85/142) showed a significant (*p* < 0.05) change in neuronal baseline activity after six daily exposures to MPD and three washout days, with 58% (49/85) showing a significant (*p* < 0.05) increase in firing rate. Upon rechallenge with MPD on ED10 MPD/ED1 MPD, 68% (96/142) of DRNs responded with a significant (*p* < 0.05) change in their neuronal firing rate. Among these responding neurons, an even split of 50% (48/96) showed a significant (*p* < 0.05) increase or decrease in firing rate (Table 1A, 2.5 mg/kg MPD, all).

Ninety-four DRNs’ activity was recorded from adult animals following the administration of 2.5 mg/kg MPD (Table 2A). Among these DRNs, 57% (54/94) responded with a significant (*p* < 0.05) change in their neuronal firing rate at ED1 MPD/ED1 BL, with the majority (91%, 49/54) exhibiting a significant (*p* < 0.05) increase in firing rate. Of these DRNs, 71% (67/94) showed a significant (*p* < 0.05) change in ED10 BL/ED1 BL, with 52% (35/67) showing an increase in firing rate. Following MPD rechallenge on ED10 MPD/ED1 MPD, 79% (74/94) responded with a significant (*p* < 0.05) change in their neuronal activity. Among these DRNs, the majority (61%, 45/74) responded with a significant (*p* < 0.05) increase in neuronal activity (Table 2A, 2.5 mg/kg MPD, all groups).

### 2.19. Impact of 2.5 mg/kg MPD on DRN Activity Recorded in Animals Expressing Behavioral Sensitization to Chronic 2.5 mg/kg MPD

Sixty-two DRNs were recorded from young animals that demonstrated behavioral sensitization to chronic exposure to 2.5 mg/kg MPD (Table 1B). Among these DRNs at ED1 MPD/ED1 BL, 89% (55/62) responded with a significant (*p* < 0.05) change in firing rate, with 89% (49/55) responding with an increase in their firing rate. When comparing ED10 BL/ED1 BL, 89% (55/62) of DRNs showed a significant (*p* < 0.05) change in firing rate. Among these neurons, the majority (80%, 49/55) showed a significant increase in firing rate. Upon rechallenge with MPD on ED10 MPD/ED1 MPD, 95% (59/62) of DRNs responded with a significant (*p* < 0.05) change in firing rate. Among these neurons, the majority (81%, 48/59) responded with an increase in firing rate (Table 1B, 2.5 mg/kg MPD, sensitized group). 

Forty-two DRNs were recorded from adult animals that demonstrated behavioral sensitization to chronic exposure to 2.5 mg/kg MPD (Table 2B). Among these DRNs at ED1 MPD/ED1 BL, 43% (18/42) responded with a significant (*p* < 0.05) change in firing rate, with 94% (17/18) responding with an increase in firing rate. Eighty-three percent (35/42) of DRNs demonstrated a significant (*p* < 0.05) change in baseline on ED10 BL/ED1 BL. Among these neurons, 63% (22/35) exhibited a decrease in their firing rate and 37% (13/35) exhibited an increase in their firing rate. Eighty-eight percent (37/42) of DRNs responded with a significant (*p* < 0.05) change in their neuronal firing rate upon rechallenge with MPD on ED10 (ED10 MPD/ED1 MPD). Among these neurons, 54% (20/37) showed a significant (*p* < 0.05) increase in firing rate (Table 2B, 2.5 mg/kg MPD, sensitized group).

### 2.20. Impact of 2.5 mg/kg MPD on DRN Activity Recorded in Animals Expressing Behavioral Tolerance to Chronic 2.5 mg/kg MPD

Eighty DRNs were recorded from young animals demonstrating behavioral tolerance to chronic 2.5 mg/kg MPD (Table 1C). Of these neurons at ED1 MPD/ED1 BL, 63% (50/80) responded with a significant (*p* < 0.05) change in firing rate following acute injection of MPD, with 72% (36/50) responding with an increase in firing rate. When comparing ED10 BL/ED1 BL, 51% (41/80) showed a significant (*p* < 0.05) change in firing rate. Among these DRNs, 46% (19/41) exhibited an increase in their ED10 BL, while 54% (22/41) showed a decrease in firing rate on ED10 BL/ED1 BL. Upon rechallenge of MPD on ED10 MPD/ED1 MPD, 61% (49/80) responded with a significant (*p* < 0.05) change in firing rate. Among these neurons, 76% (37/49) responded with a significant decrease in firing rate (Table 1C, 2.5 mg/kg MPD, tolerance group).

Fifty-two DRNs were recorded from adult animals demonstrating behavioral tolerance to chronic 2.5 mg/kg MPD (Table 2C). Of these DRNs at ED1 MPD/ED1 BL, 71% (37/52) responded with a significant (*p* < 0.05) change in neuronal firing rate, with the majority, 89% (33/37), responding with an increase in firing rate. When comparing ED10 BL/ED1 BL, 62% (32/52) displayed a significant (*p* < 0.05) difference in ED10 BL. Among these neurons, 66% (21/32) displayed an increased BL neuronal firing rate. Upon rechallenge of MPD on ED10 MPD/ED1 MPD, 71% (37/52) of DRNs responded with a significant (*p* < 0.05) change in firing rate. Of these DRNs, 62% (23/37) responded with an increase in firing rate (Table 2C, 2.5 mg/kg, tolerance group).

### 2.21. Impact of 10.0 mg/kg MPD on DRN Activity Was Recorded from All the Animals

One hundred and sixty-eight DRNs were recorded from young rats (Table 1A). Of these neurons at ED1 MPD/ED1 BL, 86% (145/168) responded with a significant (*p* < 0.05) change in neuronal firing rate activity, with 55% (80/145) demonstrating an increase in ED1 MPD/ED1 BL firing rate. When comparing ED10 BL/ED1 BL activity, 80% (135/168) of DRNs showed a significant (*p* < 0.05) change in their neuronal baseline on ED10 BL. Among these neurons, 53% (72/135) showed a significant (*p* < 0.05) increase in firing rate. Upon rechallenge of MPD on ED10 MPD/ED1 MPD, 83% (139/168) neurons responded with a significant (*p* < 0.05) change in neuronal firing rate. Among these responding neurons, 53% (73/139) responded with a significant (*p* < 0.05) increase in firing rate (Table 1A, 10.0 mg/kg MPD, all groups).

One hundred and thirteen DRN activities were recorded following 10.0 mg/kg MPD administration from all adult animals (Table 2A). Of these DRNs at ED1 MPD/ED1 BL, 73% (83/113) responded with a significant (*p* < 0.05) change in the neuronal firing rate, with the majority, 87% (72/83), exhibiting a significant (*p* < 0.05) increase in firing rate. Of the 113 DRNs recorded at ED 10 BL/ED1 BL after six daily MPD exposures and three washout days, 87% (98/113) of DRNs showed a significant (*p* < 0.05) change in their ED10 BL/ED1 BL, with the majority, 62% (61/98), exhibiting a decrease in their ED10 BL, while 38% (37/98) showed an increase in their ED10 BL/ED1 BL firing rate. Following MPD rechallenge on ED10 MPD/ED1 MPD, 89% (100/113) of DRNs responded with a significant (*p* < 0.05) change in their neuronal activity. Among these responding DRNs, the majority, 61% (61/100), responded with a significant (*p* < 0.05) decrease in their neuronal activity (Table 2A, 10.0 mg/kg MPD, all groups).

### 2.22. Impact of 10.0 mg/kg MPD on DRN Activity Recorded in Animals Expressing Behavioral Sensitization to Chronic 10.0 mg/kg MPD

One hundred and nine DRNs were recorded from young animals that demonstrated behavioral sensitization to chronic exposure to 10.0 mg/kg MPD (Table 1B). Of these DRNs, at ED1 MPD/ED1 BL, 95% (103/109) responded with a significant (*p* < 0.05) change in firing rate, with 70% (73/109) demonstrating an increase in firing rate. When comparing ED10 BL/ED1 BL, 83% (90/109) of neurons showed a significant (*p* < 0.05) change in firing rate. Of these DRNs, the majority, 59% (53/90), showed a significant increase in firing rate. Upon rechallenge of MPD on ED10 MPD/ED1 MPD, 94% (102/109) of DRNs responded with a significant (*p* < 0.05) change in firing rate. Of these neurons, 66% (67/102) responded with an increase in firing rate (Table 1B, 10.0 mg/kg MPD, sensitized group).

Twenty DRNs were recorded from adult animals that demonstrated behavioral sensitization to chronic exposure to 10.0 mg/kg MPD (Table 2B). Of these DRNs at ED1 MPD/ED1 BL, 75% (15/20) responded with a significant (*p* < 0.05) change in their firing rate; all the 15 neurons demonstrated an increase in firing rate. At ED10 BL/ED1 BL, 95% (19/20) of DRNs demonstrated a significant (*p* < 0.05) change in their neuronal firing rates. Of these 19 DRNs, 79% (15/19) exhibited a decrease in their ED10 BL activity, while 21% (4/19) exhibited an increase in their firing rates. Comparing ED10 MPD/ED1 MPD, 95% (19/20) of DRNs responded with a significant (*p*< 0.05) change in their neuronal firing rates. Of these neurons, 16% (3/19) responded with a significant (*p* < 0.05) increase in their firing rate, and 84% (16/19) responded to MPD rechallenge with a significant (*p* < 0.05) decrease in their neuronal activity (Table 2B, 10.0 mg/kg MPD, sensitized).

### 2.23. Impact of 10.0 mg/kg MPD on DRN Activity Recorded in Animals Expressing Behavioral Tolerance to Chronic 10.0 mg/kg MPD

Fifty-nine DRNs were recorded from young animals demonstrating behavioral tolerance to chronic 10.0 mg/kg MPD (Table 1C). Of these neurons at ED1 MPD/ED1 BL, 65% (45/59) responded with a significant (*p* < 0.05) change in firing rate, with 24% (11/45) responding with an increase in firing rate and the majority, 76% (34/45), with a decrease in their firing rate. When comparing ED10 BL/ED1 BL, 68% (40/59) showed a significant (*p* < 0.05) change in firing rate. Of these neurons, 55% (22/40) showed an increase in firing rate. Upon rechallenge of MPD on ED10 MPD/ED1 MPD, 58% (34/59) responded with a significant (*p* < 0.05) change in their firing rate. Of these DRNs, 18% (6/34) responded with a significant increase in firing rate, and the other 82% (23/34) responded with a significant (*p* < 0.05) decrease in firing rates, as compared to the initial MPD effects (Table 1C, 10.0 mg/kg MPD, tolerance group). 

Ninety-three DRNs were recorded from adult animals demonstrating behavioral tolerance to chronic 10.0 mg/kg MPD (Table 2C). Of these neurons at ED1 MPD/ED1 BL, 74% (69/93) responded with a significant (*p* < 0.05) change in their neuronal firing rate, with a majority, 86% (59/69), responding with an increase in firing rate. When comparing ED10 BL/ED1 BL, 84% (78/93) of DRNs displayed a significantly (*p* < 0.05) different baseline. Of these DRNs, 41% (32/78) displayed an increased ED10 BL neuronal firing rate and 54% (46/78) exhibited a decrease in their ED10 BL. Upon rechallenge of MPD on ED10 MPD/ED1 MPD, 87% (81/93) of DRNs responded with a significant (*p* < 0.05) change in firing rate when compared to responses from acute injection of MPD. Of these neurons, the majority, 56% (45/81), responded with a significant (*p* < 0.05) decrease in their firing rate, and 44% (36/81) responded with an increase in firing rate (Table 2C, 10.0 mg/kg, tolerance).

### 2.24. Response Direction (Increase or Decrease) of DRNs to MPD (Figure 3)

**Figure 3:**
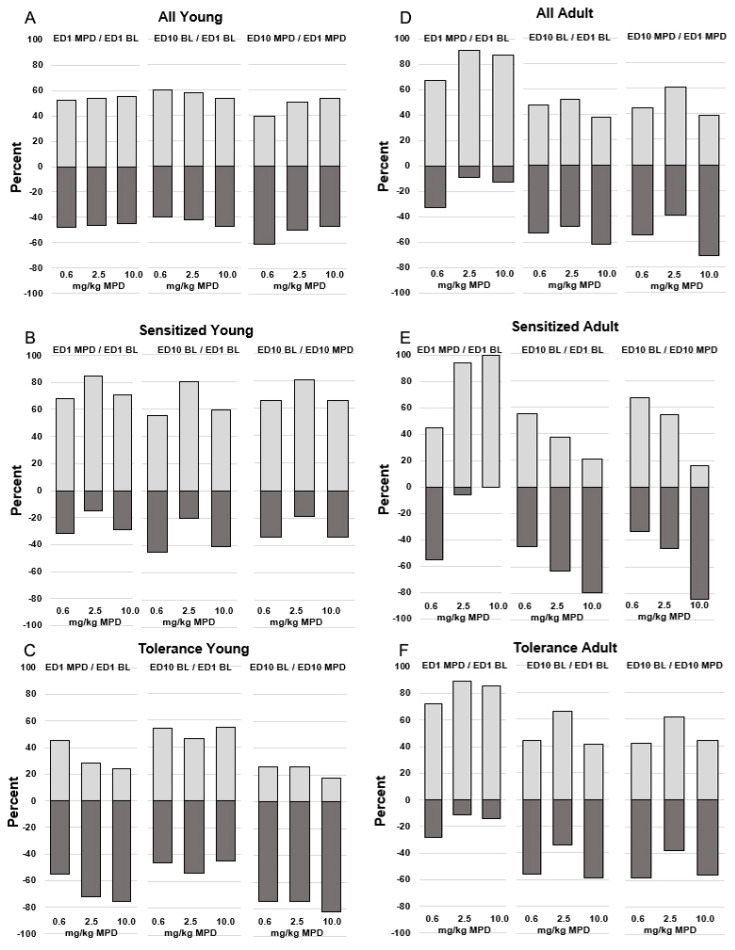
The figure summarizes the responsiveness direction (increase or decrease in % of how many DRNs respond significantly to acute and chronic MPD doses). Each segment has three columns and three sections showing in percentage how many DRNs respond significantly by either increasing or decreasing firing rates in response to acute MPD (ED1 MPD/ED1 BL), the BL change in ED10 compared to ED1 after six daily MPD exposures and three washout days (ED10 BL/ED1 BL), and the chronic effect of the drug on ED10 (ED10 MPD/ED1 MPD). In (**A**,**D**) are the DRNs recorded from all the animals. In (**B**,**E**) are the DRNs recorded only from behaviorally sensitized animals, and in (**C**,**F**) are the DRNs recorded from only the behaviorally tolerant young and adult animals.

Figure 3 consists of histograms illustrating the percentages of DRNs recorded from young and adult animals responding to each MPD dose by either significantly increasing or decreasing their neuronal activity (Figure 3). The left side of Figure 3A–C represents responses from young animals, while the right side of Figure 3D–F represents responses from adult animals.

In Figure 3A,D, percentages of DRNs that significantly (*p* < 0.05) responded to acute doses of 0.6, 2.5, and 10.0 mg/kg MPD are depicted in the left column of each figure. The middle three columns represent the changes in ED10 BL compared to ED1 BL (ED10 BL/ED1 BL), and the right column of each Figure 3A–F compares ED10 MPD to ED1 MPD (ED10 MPD/ED1 MPD).

In Figure 3A–C, the young rats group demonstrates significant ((F 2,41) = 9.573, *p* < 0.05) differences among the three young groups (all, sensitized, and tolerant), as well as among the different MPD doses (0.6, 2.5, and 10.0 mg/kg MPD). By segregating the “all” group into DRNs recorded from behaviorally sensitized animals or behaviorally tolerant animals, it becomes apparent that neuronal responses to MPD from behaviorally sensitized animals significantly ((F 2,42) = 4781, *p* < 0.05) differ from those recorded from behaviorally tolerant animals. Similar trends are observed in the adult animal groups (Figure 3D–F).

When comparing responses between young and adult groups overall (Figure 3A,D), no significant differences in response direction are observed between the ages. However, significant ((F 2,42) = 4692, *p* < 0.05) differences in response directions (increase or decrease) to 0.6, 2.5, and 10.0 mg/kg MPD are noted when comparing recordings obtained from animals expressing behavioral sensitization (Figure 3B,E) to those expressing behavioral tolerance (Figure 3C,F). These observations suggest that the accurate assessment of MPD’s effects requires evaluating neuronal recordings based on the animal’s behavioral response to repetitive (chronic) drug exposure. NOS and TD activity responded similarly to NOM.

## 3. Discussion

The objective of this study was to investigate whether the acute and chronic dose–response effects of MPD on young rats is the same or different than the effects of MPD on adult male rats using concomitant behavioral and neuronal recording from the dorsal raphe (DR) area. Each animal in this study was also evaluated independently based on their behavioral response to repetitive (chronic) MPD exposure as compared to the initial (acute) response to MPD to determine whether they express behavioral sensitization or tolerance.

The main findings of this study are as follows: (1) The same chronic dose of 0.6, 2.5, and 10.0 mg/kg MPD elicits behavioral sensitization in some animals and behavioral tolerance in others. (2) Significant age differences in response to MPD were observed in the ratio of how many young compared to adult rats expressed behavioral sensitization or behavioral tolerance to chronic MPD as compared to the initial (acute) MPD effects. (3) The ratio of DRN responses to acute and chronic 0.6, 2.5, and 10.0 mg/kg MPD had similar response directions in all the young and all the adult rat groups, in terms of total responsiveness: how many DRNs responded to MPD with either excitation or attenuation in their firing rate. However, when the DRN responses were evaluated based on the animal’s behavioral responses to chronic MPD and neuronal recordings obtained from the animals that exhibit behavioral tolerance were separately divided from the recordings from animals that expressed behavioral sensitization into their own groups, the DRN recordings from these two age subgroups had significantly different ratios of how many DRNs responded by excitation or attenuation of the firing rates when comparing to the acute and chronic MPD administration from both age groups. (4) DRNs recorded from animals exhibiting behavioral tolerance to chronic MPD treatment are significantly more likely to respond to MPD with decreases in their firing rates as compared to the initial (acute) MPD effect, while DRNs recorded from animals expressing behavioral sensitization tended to respond to chronic MPD by further increasing their firing rates as compared to the acute MPD effect. However, significant differences between the two age groups were observed in the percentage of how many DRNs responded to each acute and chronic MPD dose as well the behavioral responses to MPD. This observation indicates that the effect of MPD on animals’ behaviors and on DRNs in young animals is significantly different from the effect of MPD on DR neurons recorded from adult animals. In addition, this observation supports our hypothesis that the same chronic dose of 0.6, 2.5, and 10.0 mg/kg MPD will elicit behavioral sensitization in some animals and behavioral tolerance in others and that there is a correlation between the DRN response to chronic MPD and the behavioral expression to chronic MPD. Moreover, the observation indicates that it is essential to evaluate drug effects on the brain based on the chronic effects of the drug on the subject’s behavior.

Most of the studies investigating the properties of MPD have focused mainly on MPD’s effects on the DA system and some of the NE system. There are limited studies that suggest that serotonin signaling participates in MPD action or in addiction, vulnerability, and cravings [22,30,32,33,49,54]. During development, i.e., in young, overproduction of synaptic connections and receptors occurs, followed by pruning or competitive elimination [41,42,55,56]. At therapeutic dosages, MPD has been shown to alter the expression of dopaminergic regulator genes in the sensorimotor striatum, a region also involved in learning and habit formation [57]. 5-HT reuptake inhibitors have been shown to potentiate MPD-induced gene regulation in the young striatum.

We propose the following mechanism of MPD action: (1) Following acute MPD exposure, MPD binds to DA, NE, and 5-HT transporters, preventing the reuptake of DA, NE, and 5-HT from the synaptic cleft into the presynaptic terminals [51,52]. This increases the DA, NE, and 5-HT levels in the synaptic cleft leading to increases in locomotion and neuronal activities [19]. MPD is metabolized, the DA, NE, and the 5-HT levels return to pre-MPD levels, and the animals’ behavioral expression and neuronal firing rates return to baseline levels [58,59,60]. (2) Repeated MPD exposure has been associated with plasticity in the mesocorticolimbic system [59,60]. Molecular and cellular plasticity in the brain requires changes in gene expression. Gene expression is controlled by a series of DNA-binding proteins known as transcription factors. Several transcription factors have been implicated in this regulation, such as CREB and delta Fos B [59,61].

Significant differences were observed between the age groups following chronic MPD exposure to 0.6 and 2.5 mg/kg MPD. These different effects between young and adult rats may be explained by age differences in the upregulations of transcription factors that lead to different levels of protein synthesis [60]. The age differences can also be explained by varying plasticity processes that are ongoing in young animals as compared to adult animals. To explain why some animals respond to chronic MPD with behavioral sensitization and others with behavioral tolerance, we can assume that MPD-elicited sensitization is the result of extracellular signal related to Kinase phosphorylation (ERK) that leads to increased levels of delta Fos B, AMPA receptor subunits, and ionotropic transmembrane glutamate receptors that mediate synaptic transmission in the central nervous system and subsequent increases in the neuronal and behavioral activities [48,59,61]. While in behaviorally tolerant animals, the same chronic MPD exposure results in the upregulation of cAMP response element-binding protein (CREB), which leads to decreases in AMPA receptor subunits and decreases in neuronal and behavioral activities [59,61]. The reason why MPD in some animals elicits upregulation in CREB and in delta Fos B needs further studies.

The DR also contains GABAergic inhibitory neurons as well as glutamatergic, dopaminergic, and substance P-containing neurons [62]. It was reported that the DR serotonergic neurons exhibit moderate regular firing patterns and exhibit a long duration action potential as compared to non-serotonergic DR neurons [62,63,64]. Based on the moderate firing rates and the neural spike duration that was used to sort and analyze the recorded spikes, it is likely that most of the DRNs recorded in this study were serotonergic.

Immunohistochemistry studies have demonstrated that withdrawal from chronic MPD treatment results in the upregulation of serotonin transporter (SERT) and consequently SERT density, thus suggesting that 5-HT may play a part in participating in the long-term potentiation following repetitive MPD exposure [65]. This observation indicates that DRNs recorded from young were more sensitive to MPD than the DR neurons recorded from adult animals, expressed mainly by the ED10 BL/ED1 BL neuronal activity that was significantly higher in the recordings obtained from young animals as compared to those DRNs recorded from adult animals. Since the modulation of ED10 BL/ED1 BL indicates withdrawals, it raises concerns that MPD use in young increases potential for abuse as compared to adults since behavioral withdrawal is one of the experimental biomarkers indicating that a drug has the characteristics and the potential to elicit dependence [19,48,66].

While the dopaminergic and glutaminergic signals have definitive roles in the neuroplasticity underlying addiction, there are limited studies of the serotonergic signals that participate in the expression of drug dependence [49]. Additional support of the role of 5-HT in the expression of drugs of abuse is provided by Sora et al. [67]. In these studies, mice with genetically deleted DA and NE transporters show a continued response to the reward properties of drugs of abuse, such as cocaine, as evidenced by self-administration and a conditioned place preference experimental model [67,68], indicating that non-CA mechanisms also contribute to psychostimulant effects.

Salman et al. [69] reported that 2.5 mg/kg MPD exposure increases the metabolism of 5-HT and improves performance in water maze tests, which suggests that the significant therapeutic effects of MPD include increasing 5-HT in the synaptic cleft and reducing GABA-a receptor mRNA expression to release excitatory glutamate from the inhibitory influence of GABA. We agree with Kirby et al. [49] and Ruocco et al. [70] that the serotonergic signals play a role in psychostimulant action, as evidenced by our observations that chronic MPD exposure elicits withdrawal, sensitization, and tolerance.

Can six daily MPD exposures be considered as chronic effects of the drug? In our opinion—yes—based on the following: The life expectancy of the average male human in Europe and the USA before the 2020 COVID-19 pandemic was about 78 years. Therefore, 78 × 360 days/years = 28,080 days = 100%. Rat life expectancy is 2 years = 100%. Six days of a rat’s life is 1.7%. Furthermore, 1.7% of a human’s life is 477 days or 15.9 month or 1.3 years. In our opinion, 1.3 years of daily treatment can be considered as chronic treatment [71].

In conclusion, the main findings indicate that MPD affects DR neurons, the source of serotonin to the CNS, in a dose-dependent manner. This suggests that one of the therapeutic effects of MPD is its impact on the DR and serotonin modulation. Furthermore, this study reveals significant age-related differences in response to MPD, underscoring the importance of evaluating MPD effects across different age groups based on their behavioral responses to both chronic and initial MPD exposure. These differences hold clinical significance. Additionally, the observation that some animals exhibit behavioral sensitization while others show behavioral tolerance to chronic MPD underscores the need for the individualized assessment of responses to the drug’s chronic effects. The presence of sensitization and tolerance serves as an experimental biomarker, indicating the potential for drug abuse.

## 4. Materials and Methods

### 4.1. Subjects

Young male Sprague Dawley rats at post-natal day 30 and adult male rats at post-natal day 50 were obtained from Harlan (Indianapolis, IN, USA). Prior to DR electrode implantation, the rats underwent an acclimation period of 3–5 days. They were provided ad libitum access to food and water and housed under standard enriched laboratory conditions. The ambient temperature was maintained at 21 ± 2 °C with a relative humidity of 37–45%, following a 12 h:12 h alternating light–dark cycle with lights on at 6:00. All animal procedures were conducted with the approval of the University of Texas Health Science Center Animal Welfare Committee (AWC-12-032) and were in accordance with the National Institute of Health Guide for Care and Use of Laboratory Animals.

### 4.2. Surgical Procedure

Young animals were anesthetized with a 30 mg/kg pentobarbital intraperitoneal (i.p.) injection, while adults rats received a 50 mg/kg pentobarbital i.p. injection. Prior to surgery, each rat’s head was shaved, and lidocaine hydrochloride topical gel was applied to the shaved area for local anesthesia. Subsequently, the animal was positioned in a stereotaxic Grass head holder instrument, and an incision was made to expose the skull. Bilateral holes were drilled in the skull above the DR nucleus (DRN) at 0.2 lateral to the midline and 7.0 mm posterior from the Bregma for young rats and 0.2 lateral from the midline and 7.8 mm posterior to the bregma for adults rats, using the Sherwood and Timiras adolescent rat brain atlas [72] for young rats and the Paxinos and Watson rat brain atlas [73] for adult rats. An additional hole was made in front of the frontal sinus for the reference electrode. To secure the skull cap, six anchor screws were inserted into vacant spots on the skull, and dental acrylic cement was applied. Two twisted nickel–chromium wires (insulated except at tips), measuring 60 µm in diameter, were each connected to 1 cm copper connector pins and inserted into the DRN in each hemisphere, resulting in four recording electrodes per animal. Neuronal activity was monitored during electrode placement. The electrodes were inserted to a depth of 6 mm and, upon detecting satisfactory neuronal activity, were secured to the anchor screws and skull with dental acrylic cement. Electrodes failing to detect satisfactory activity were lowered in 5 to 10 µm increments until satisfactory neuronal activity with a 3:1 signal-to-noise ratio was achieved [51,52,53]. Animals were allowed to recover from surgery for 5 to 7 days.

### 4.3. Drug

Methylphenidate hydrochloride (MPD) was obtained from NIDA. Previous experiments utilizing dose–response MPD protocols ranging from 0.1 to 40.0 mg/kg i.p. indicated behavioral effects of MPD at doses of 0.6 mg/kg and higher (Gaytan et al., 1996) [74]. Therefore, MPD was administered at doses of 0.6, 2.5, and 10.0 mg/kg, corresponding to low, moderate, and high experimental dosages, respectively. MPD was dissolved in a 0.9% isotonic saline solution for i.p. injection. Control subjects received injections of 0.8 mL isotonic saline solution (0.9% NaCl). All MPD injections were titrated to a volume of 0.8 mL with 0.9% saline to ensure consistent injection volumes across all animals.

### 4.4. Experimental Protocol and Data Acquisition

The neuronal and locomotor behavioral activity of the animals started at P-40 for young rats and P-60 for adults (Table 3. A wireless neuronal recording system (TBSI, Durham, NC, USA) and an open-field computerized animal activity system (Accuscan, Columbus, OH, USA) for locomotor behavioral data acquisition were used. The TBSI head stage was affixed to the rat’s head cap, housing the electrode pins, which transmitted electrical signals from the electrodes to a remote receiver connected to an analog-to-digital converter (Micro 1401-3; Cambridge Electronic Design (CED) England). Neuronal activity from each electrode was stored on a PC using the CED Spike 2.7 software.

The open-field system comprised a 40 × 40 × 32 cm cage with 16 × 16 infrared beams positioned 5 and 8 cm above the cage floor [51,52,53,74], detecting interruptions in the infrared beams caused by animal movement at a frequency of 100 Hz. The interruptions were then compiled by the Oasis software 5 (Accuscan, Columbus, OH, USA) and downloaded to a PC every 10 min. The software categorized beam interruptions into three locomotive behaviors: the number of movements (NOM), total distance traveled (TD) in centimeters, and the number of stereotypic movements (NOS), defined as repetitive movements with at least one-second intervals between them (Figure 1A). Data were recorded for 60 min post injection of either saline or MPD on experimental day (ED) 1 and ED10 (Figure 1B). This behavioral recording aimed to differentiate between animals exhibiting behavioral sensitization following repetitive (chronic) MPD exposure and those showing behavioral tolerance compared to the initial MPD effect [15,19,66]. The locomotor behavioral data served as the basis for analyzing the neuronal recordings. Since some rats of both age groups displayed behavioral sensitization and others showed behavioral tolerance to each of the chronic 0.6, 2.5, and 10.0 mg/kg doses of MPD, we divided the animals into three groups for data evaluation: (1) data obtained from all animals—all groups; (2) data obtained only from animals expressing behavioral sensitization—sensitized group; and (3) data obtained only from animals expressing behavioral tolerance—tolerant group.

The experiment lasted for 11 days (Table 3. Rats of each age were randomly subdivided into four groups: saline (control), 0.6, 2.5, and 10.0 mg/kg MPD treatment groups. On ED1, rats were placed within their home cage in a Faraday testing cage to reduce background noise. The wireless head stage was connected to the electrode pins of the skull cap, and animals were allowed to acclimate for an additional 20 to 30 min before the recording session. On ED1, neuronal and behavioral activity was recorded concurrently for one hour each following an initial injection of 0.8 mL saline; these data served as a baseline (BL) activity (ED1 BL control). The animals then received a second injection of either saline or 0.6, 2.5, or 10.0 mg/kg MPD. From ED2 through ED6, animals received either saline or MPD injections in their home cage without behavioral or neuronal recordings to induce chronic MPD effects. This was followed by a three-day washout period from ED7 through ED9 where no injections were administered. On ED10, animals received a 0.8 mL saline injection to be used as ED10 BL neuronal and behavioral activity, and recording resumed for 60 min. Subsequently, the animals were rechallenged with either saline, 0.6, 2.5, or 10.0 mg/kg MPD (ED10 Sal or ED10 MPD), and an additional 60 min of recording was conducted similar to that on ED1 (Table 3) and [19,66].

### 4.5. Behavioral Data Analysis

The acquired data were utilized for three primary comparisons: (1) Acute MPD effects: locomotor activity following acute MPD administration on ED1 was compared against baseline activity post saline administration on ED1 (ED1 MPD/ED1 BL), aiming to discern the immediate impacts of MPD. (2) Chronic MPD effects on BL activities: locomotor activity post saline injection on ED10 was compared to the activity post saline injection on ED1 (ED10 BL/ED1 BL) to identify any significant alterations in baseline locomotor activity following six days of chronic MPD exposure, followed by three washout days, aiming to discern withdrawal. (3) Behavioral sensitization or tolerance: Locomotor activity after MPD administration on ED10 was compared to that on ED1 (ED10 MPD/ED1 MPD) to determine chronic effects and ascertain whether behavioral sensitization or tolerance had developed. Animals exhibiting significantly increased locomotor activity upon MPD rechallenge on ED10 compared to behavioral activity of MPD administration on ED1 were deemed to display behavioral sensitization, while those showing a significant decrease were considered to display behavioral tolerance.

To assess the effect of MPD on individual animals, both the Student’s *t*-test and the Critical Ratio (CR) test were employed. CR = (E − C)/√(E + C) = ±1.96; a value above +1.96 indicates a significant increase in activity, while a value below −1.96 indicates a significant decrease after MPD administration. In the case of acute MPD effects, E denotes activity count after MPD injection on ED1, while C represents the activity after saline (control) injection on ED1 (ED1 MPD/ED1 BL). For repeated injection (chronic) effects, E represents activity following Sal or MPD injection on ED10, while C represents activity after Sal or MPD dose on ED1 (ED10 BL/ED1 BL and ED10 MPD/ED1 MPD).

Individual rats were then categorized based on their exhibited behavioral sensitization or tolerance and subsequently sorted accordingly. To determine significant differences among MPD doses and age groups, a two-way ANOVA was conducted, with a *p*-value of <0.05 considered the threshold for significance.

### 4.6. Neuronal Data Analysis

**Figure 4:**
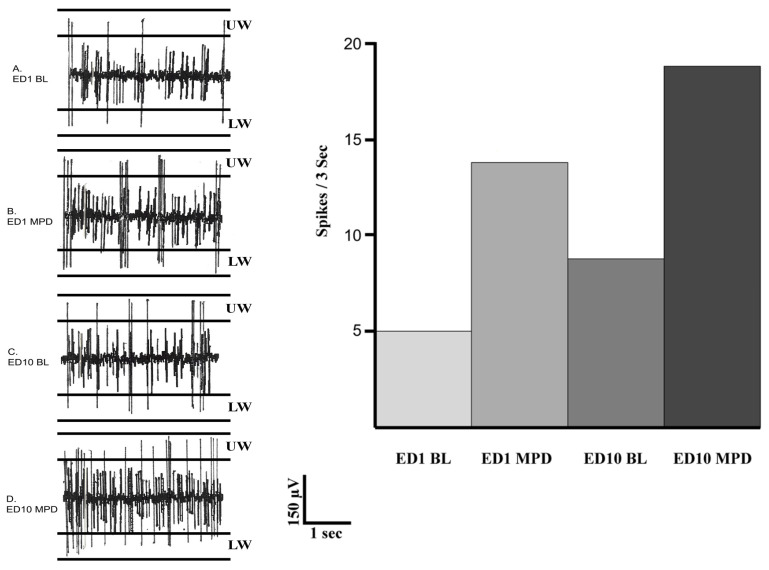
The figure shows 4 traces of analog recordings on the left side. Upper trace Baseline recordings on experimental day 1 (ED1 BL). One below the upper trace Recordings following 2.5 mg/kg MPD on ED1 showing that ED1 MPD elicits excitation as compared to ED1 BL. Two below the upper trace ED10 BL recording after six daily 2.5 mg/kg MPD and three washout days showing withdrawal activity. The lowest trace Recording following repeated 2.5 mg/kg MPD on ED10 showing MPD sensitization as compared to ED1 MPD. The figure shows typical neuronal recordings and the upper (UW) and lower (LW) windows that use the first stage of spike sorting. On the right is a histogram summarizing the number of spikes in the 4 traces showing the MPD effects.

CED Spike 2.7 software was used for spike sorting and statistical analysis of neuronal data. Neuronal activity processing employed both low- and high-pass filters ranging from 0.3 to 3.3 kHz. In addition, two window discriminator levels were used, distinguishing positive- and negative-going spikes (Figure 4). Spikes were extracted based on predefined amplitude windows, with peak amplitudes falling within the range triggering a template creation process. A template, comprising selected spikes exhibiting durations of 0.8 to 1.2 milliseconds, was generated utilizing a thousand waveform data points. This algorithmic approach enabled the extraction of high-dimensional reference points from templates, facilitating robust spike sorting even in the presence of noise, false threshold crossings, and waveform overlaps. Each incoming spike waveform was compared against temporal templates to identify the best fitting template, minimizing residue variance. Waveforms deviating beyond an 80% threshold from the template were rejected, ensuring a spike sorting accuracy of approximately 95%. To ensure consistency, data evaluation of DRN activity recorded on ED1 and ED10 used the same sorting parameters, and the same templates were applied to both ED1 and ED10 files for sorting ED1 and ED10 activity, guaranteeing identical waveform sorting parameters between sessions.

**Figure 5:**
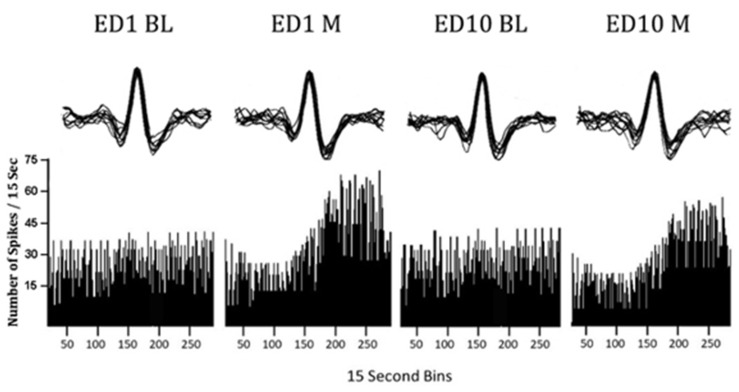
A histogram of DR neurons (DRNs) recorded from adult rats summarizing 60 min sequential neuronal firing rates following acute and chronic 2.5 mg/kg MPD exposure. The first panel, ED1 BL, shows the DRN activity recorded at baseline on ED1. The second panel, ED1 MPD, shows the DRN activity recorded after acute 2.5 mg/kg MPD exposure. The third panel, ED10 BL, shows the DRN activity recorded after previous exposure to six daily MPD exposures and three washout days to determine if there is a withdrawal response. The fourth panel, ED10 MPD, shows DRN activity after chronic MPD administration to determine whether sensitization or tolerance occurred. Above each histogram are 20 random superimposed spikes sorted during the 60 min recording session to produce the histograms, aiming to demonstrate that the same DRN pattern was counted during each 60 min recording session.

Following spike sorting, data were exported to a spreadsheet to calculate the average neuronal firing rates per treatment and generate sequential firing rate histograms (Figure 5). Statistical comparisons were conducted for DRN activity across various conditions: (1) Comparison between DRN activity post initial MPD exposure and BL activity following saline administration on ED1 to ascertain acute MPD effects (ED1 MPD/ED1 BL). (2) Comparison of baseline DRN activity on ED10 BL with that on ED1 BL to detect expression of withdrawal after six daily MPD exposures and three washout days (ED10 BL/ED1 BL). (3) Evaluation of DRN activity post MPD administration on ED10 versus activity following initial MPD exposure on ED1 to determine chronic MPD effects (ED10 MPD/ED1 MPD; sensitization or tolerance). The significance and direction of changes in neuronal firing rates for each DRN were assessed using the Student’s *t*-test and the Critical Ratio (CR) test. A CR value above +1.96 indicated a significant (*p* < 0.05) increase in activity, while a value below −1.96 indicated a significant (*p* < 0.05) decrease after MPD administration. Based on this calculation, the neurophysiological data analysis was categorized into three subgroups: (1) All group: electrophysiological data recorded from all animals. (2) Sensitized group: neuronal data recorded from animals displaying behavioral sensitization. (3) Tolerance group: neuronal data recorded from animals exhibiting behavioral tolerance. Differences in firing rates among these subgroups were analyzed using the chi-square test, with *p* < 0.05 indicating significance.

### 4.7. Statistical Analysis

All statistical tests and analyses are performed in R 4.0.5. The chi-square test was used to examine for significant differences in the percentages of neuronal units responding to MPD among the six brain regions. If the chi-square test result indicated that the percentage of responding neuronal units was significantly different among the six brain regions, with a *p*-value of less than 0.05, the logistic regression model was then used to fit the data and post hoc comparisons were performed to identify the regions(s) with significantly different response rates compared to other regions. The post hoc comparisons were conducted with the function “contrast” in the R package “emmeans”. The Bonferroni correction method was used to adjust *p*-values for multiple comparisons. The ratio of neuronal units responding to MPD was also analyzed with increased vs. decreased firing rates among six different brain regions using the chi-square test. If there were significant differences among the different brain regions, post hoc comparisons were performed to identify the region(s) with significantly different ratios of neuronal units responding to MPD with increased vs. decreased firing rates when compared to other regions.

### 4.8. Histological Verification of Electrode Placement

On ED 11, animals were administered an overdose of sodium phenobarbital. Subsequently, they underwent intracardial perfusion with a 10% formaldehyde solution containing 3% potassium ferrocyanide. A 20 µA current was then applied through the electrode pin for 20 s to induce a small lesion at the recording sites in the DR electrode tip. The brain was subsequently removed and preserved in 10% formalin for histological processing. Electrode placement in the DRN was confirmed by the presence of the lesion site and Prussian blue spot, with reference to the young rat brain atlas [72] for young rats and the rat brain atlas [73] for adult rats.

## 5. Conclusions

When the data from all the animals were divided into data obtained from behavioral sensitization animals separated from data obtained from behavioral tolerance group animals based on behavioral recordings for chronic MPD as compared to initial MPD effects, it was found that the DRNs recorded from behaviorally sensitized animals exhibited significant (*p* < 0.001) differences in response to MPD rechallenge on ED10 as compared to the DRNs recorded from behaviorally tolerant animals. Furthermore, the ratio of how many young rats as compared to adult rats express behavioral sensitization or tolerance to the same MPD dose was found to have a significant (*p* < 0.001) difference. These correlations suggest a different effect of MPD on young brain development compared to adults. The difference in response to MPD by age needs further study.

This study provides evidence that the DR and serotonergic signaling participate in the effect of MPD, and the role of serotonergic signaling in the response to a psychostimulant, i.e., serotonin (5-HT) signaling, and the DR are important in the mechanism of action of MPD therapy in behavioral disorders; in addition, the DRNs participate in the expression of withdrawal, sensitization, and tolerance to chronic MPD. This study supports Kirby et al. [49] and Ruocco et al. [70] observations that serotoninergic signaling plays a role in the expression of chronic psychostimulants and that there are significant age differences in response to MPD exposure. This study provides important evidence of the role of the DR and serotonergic signaling in response to psychostimulants.

## Figures and Tables

**Table 3:** Four groups of animals were used for each age (young and adult), as follows: saline, 0.6, 2.5, and 10.0 mg/kg MPD. On experimental day 1 (ED1), animals were given an initial dose of saline and recordings were taken for one hour to obtain ED1 baseline (BL) followed by one of the four designated injections of saline, 0.6, 2.5, or 10.0 mg/kg of MPD, and recordings were resumed for an additional hour post injection. On ED 2–6, the animals were given an injection each morning of the designated dose. ED 7–9 were washout days where the animals received no injection of any kind. On ED10, the animals were given another dose of saline to obtain ED10 BL after six daily injections of either saline or MPD for one hour followed by the designated MPD dose for one hour, and recordings were taken, identical to those on ED1. *—indicates the behavioral and neuronal recording day.

		Experimental Days (ED)			
Treatment Groups		ED 1 *	EDs 2–6	EDs 7–9	ED10 *
1	Saline	Saline/Saline	Saline	Washout	Saline/Saline
2	0.6 mg/kg MPD	Saline/0.6 mg/kg MPD	0.6 mg/kg MPD	Washout	Saline/0.6 mg/kg MPD
3	2.5 mg/kg MPD	Saline/2.5 mg/kg MPD	2.5 mg/kg MPD	Washout	Saline/2.5 mg/kg MPD
4	10.0 mg/kg MPD	Saline/10.0 mg/kg MPD	10.0 mg/kg MPD	Washout	Saline/10.0 mg/kg MPD

## Data Availability

In the university server.
